# Association of acute myocardial infarction cardiac arrest patient volume and in‐hospital mortality in the United States: Insights from the National Cardiovascular Data Registry Acute Coronary Treatment And Intervention Outcomes Network Registry

**DOI:** 10.1002/clc.23146

**Published:** 2019-02-07

**Authors:** Michael C. Kontos, Christopher B. Fordyce, Anita Y. Chen, Karen Chiswell, Jonathan R. Enriquez, James de Lemos, Matthew T. Roe

**Affiliations:** ^1^ Internal Medicine (Cardiology Division), Virginia Commonwealth University Richmond Virginia; ^2^ Division of Cardiology University of British Columbia Vancouver British Columbia Canada; ^3^ Duke Clinical Research Institute Durham North Carolina; ^4^ Internal Medicine (Cardiology Division), University of Missouri‐ Kansas City and Saint Luke's Mid America Heart Institute Kansas City Missouri; ^5^ Internal Medicine (Cardiology Division), University of Texas Southwestern Medical Center Dallas Texas

**Keywords:** cardiac arrest, myocardial infarction, outcomes

## Abstract

**Background:**

Little is known about how differences in out of hospital cardiac arrest patient volume affect in‐hospital myocardial infarction (MI) mortality.

**Hypothesis:**

Hospitals accepting cardiac arrest transfers will have increased hospital MI mortality.

**Methods:**

MI patients (ST elevation MI [STEMI] and non‐ST elevation MI [NSTEMI]) in the Acute Coronary Treatment Intervention Outcomes Network Registry were included. Hospital variation of cardiac arrest and temporal trend of the proportion of cardiac arrest MI patients were explored. Hospitals were divided into tertiles based on the proportion of cardiac arrest MI patients, and association between in‐hospital mortality and hospital tertiles of cardiac arrest was compared using logistic regression adjusting for case mix.

**Results:**

A total of 252 882 patients from 224 hospitals were included, of whom 9682 (3.8%) had cardiac arrest (1.6% of NSTEMI and 7.5% of STEMI patients). The proportion of MI patients who had cardiac arrest admitted to each hospital was relatively low (median 3.7% [25th, 75th percentiles: 3.0%, 4.5%]).with a range of 4.2% to 12.4% in the high‐volume tertiles. Unadjusted in‐hospital mortality increased with tertile: low 3.8%, intermediate 4.6%, and high 4.7% (*P* < 0.001); this was no longer significantly different after adjustment (intermediate vs high tertile odds ratio (OR) = 1.02; 95% confidence interval [0.90‐1.16], low vs high tertile OR = 0.93 [0.83, 1.05]).

**Conclusions:**

The proportion of MI patients who have cardiac arrest is low. In‐hospital mortality among all MI patients did not differ significantly between hospitals that had increased proportions of cardiac arrest MI patients. For most hospitals, overall MI mortality is unlikely to be adversely affected by treating cardiac arrest patients with MI.

## 1 INTRODUCTION

Out of hospital cardiac arrest associated with acute myocardial infarction (MI) occurs in 8% to 10% of acute MI patients.^1^, ^2^, ^3^ Although there have been significant improvements in outcomes, patients with cardiac arrest continue to have a high‐mortality despite aggressive treatment, including frequent use of urgent revascularization.^1^, ^2^, ^3^, ^4^, ^5^

Risk‐adjusted mortality after MI is considered an important quality metric that influences both value‐based payments and quality rankings. Concern has been raised that conditions, such as cardiac arrest and cardiogenic shock cannot be adequately risk adjusted.^6^, ^7^, ^8^, ^9^ Thus, it is possible that centers that receive a larger number of Out of Hospital cardiac arrest (OHCA) patients may see an increase in overall risk‐adjusted MI mortality. Therefore, we investigated whether a higher cardiac arrest patient volume is associated with overall in‐hospital mortality in patients presenting with both non‐ST elevation MI (NSTEMI) and STEMI, using data from the National Cardiovascular Data Registry (NCDR) Acute Coronary Treatment Intervention Outcomes Network Registry (ACTION Registry).

## 2 METHODS

The ACTION Registry is a nationwide, ongoing, voluntary quality improvement registry sponsored by the American College of Cardiology (ACC) that focuses exclusively on patients with acute MI. Details of the design and conduct of the registry have been previously described.^10^ In brief, the registry includes consecutive patients with a primary diagnosis of STEMI or NSTEMI, who have prolonged ischemic rest symptoms occurring within 24 hours before admission or up to 72 hours for STEMI; ischemic ECG changes associated with STEMI; or positive cardiac markers associated with NSTEMI within 24 hours after initial presentation.

Definitions for the data elements of the registry are available at: https://www.ncdr.com/webncdr/action/home/datacollection. The ACTION Registry also has a data quality program, including data abstraction training, data quality thresholds for inclusion, site data quality feedback reports, independent auditing, and data validation. Auditing of data has demonstrated chart review agreement of >93% of collected variables.^11^

This registry was either approved by an institutional review board, or considered quality assurance data and not subject to institutional review board approval based on individual site determinations.^10^

### 2.1 Patient population

The initial STEMI and NSTEM population included 476 073 MI patients from April 2011 to December 2014. The following patients were excluded sequentially: patients who arrived to hospitals without surgical capabilities (n = 68 071 with 236 hospitals); patients from sites that did not submit both STEMI and NSTEMI patients (n = 139 with 1 hospital) because of biased submission; patients from hospitals that did not submit data for 15 consecutive quarters (n = 147 422 with 315 hospitals); patients who had missing cardiac arrest on first medical contact (n = 1562), and patients who transferred out to another hospital (n = 5997) as it was unable to determine the patient's in‐hospital outcomes. This resulted in a total of 252 882 patients from 224 hospitals that were included in the final analysis.

### 2.2 Definitions

Cardiac arrest was defined as patients who were evaluated by emergency medical services (EMS) or emergency department (ED) personnel and either: received attempts at external defibrillation (by lay responders or emergency personnel) or chest compressions by organized EMS or ED personnel; or were pulseless but did not receive defibrillation or cardiopulmonary resuscitation by EMS personnel. For each hospital, proportion of cardiac arrest patients was calculated as the number of cardiac arrest patients divided by number of MI patients. Sorting by hospital proportions of cardiac arrest patients, hospitals were grouped into three tertiles: low, middle, and high. Hospitals in the low tertile group had the lowest proportions of cardiac arrest patients, while hospitals in the high tertile group had the highest proportions of cardiac arrest patients. ACTION Registry in‐hospital major bleeding was defined as previously described.^12^ Given that a majority of patients undergoing coronary bypass graft surgery (CABG) receive blood transfusions related to the surgery, bleeding events were considered only if they occurred before CABG. An academic hospital was defined as membership in the council of teaching hospitals.

### 2.3 Statistical analysis

Patient baseline characteristics, treatment pattern, and hospital characteristics were stratified by tertiles of hospital cardiac arrest proportions for the overall population and among cardiac arrest patients. In addition, in‐hospital outcomes were reported for patients with and without cardiac arrest stratified by hospital cardiac arrest tertiles. Categorical variables were reported as percentages and continuous variables were reported as median (25th, 75th percentiles). *χ* ^2^ and Kruskal‐Wallis tests were used to compare categorical and continuous variables, respectively. To explore whether there was a temporal trend of the hospital proportion of cardiac arrest MI patients, median (25th, 75th percentiles) were presented across year of hospital arrival, where Kruskal‐Wallis test was used to test the differences.

To investigate the relationship between hospital cardiac arrest tertiles and in‐hospital mortality, a logistic generalized estimating equations regression with an exchangeable working correlation matrix to account for within‐hospital clustering of outcome was used.^13^ Although this working correlation structure assumes that hospitals are independent after adjusting for covariates, empirical SE estimates were used for inference, which provides robustness against possible misspecification of the correlation structure. Covariates in the model were from the previously validated and published ACTION Registry in‐hospital mortality paper,^14^ which included: age, sex, race, weight, heart failure, cardiogenic shock, systolic blood pressure, and heart rate at presentation, MI type (NSTEMI and STEMI), hypertension, diabetes mellitus, prior peripheral arterial disease, current/recent smoker, dyslipidemia, prior MI, prior percutaneous coronary intervention, prior CABG, prior heart failure, prior stroke, initial serum creatinine, initial hemoglobin, initial troponin values, home medications (aspirin, clopidogrel, warfarin, beta‐blocker, angiotensin‐converting enzyme inhibitor, angiotensin receptor blocker, aldosterone blocking agent, statin, and non‐statin lipid‐lowering agent), insurance status (health maintenance organization or private, Medicare, Medicaid, Self‐pay or none, and other), and hospital characteristics (region, academic hospitals, and total number of hospital beds).

Missing data was approximately 1% to 2% for all the covariates in the model. When modeling in‐hospital mortality, missing values in continuous covariates were imputed as sex‐specific medians of the non‐missing values. For categorical variables, missing values were imputed to the most frequent group. A *P*‐value of <0.05 was considered significant for all analyses. All statistical analyses were performed using SAS version 9.4 software (SAS Institute, Cary, North Carolina).

## 3 RESULTS

**Figure 1:**
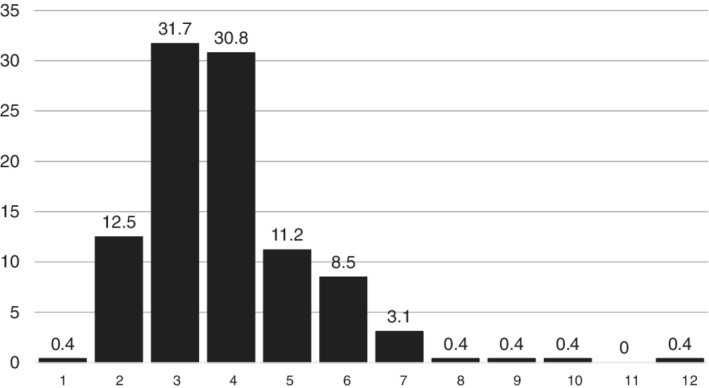
Distribution of the hospital proportion of patients with myocardial infarction with cardiac arrest across hospitals

A total of 9682 MI patients had cardiac arrest, which included 7088 patients with STEMI (73.2% of cardiac arrest patients, accounting for 7.5% of all STEMI patients) and 2594 who had NSTEMI (26.8% of cardiac arrest patients, accounting for 1.7% of NSTEMI patients). The median proportion of MI patients who had cardiac arrest admitted to each hospital was 3.7% (25th, 75th percentiles: 3.0%, 4.5%, range 0.9 to 12.4%) (Figure 1), which remained constant over the study period (median 3.7% in 2011, 3.8% 2012, 3.5% 2013, and 3.6% in 2014). When separated into tertiles, the range of hospital proportion of MI patients who had cardiac arrest in the low tertile was 0.9% to 3.2%, in the middle tertile was 3.2% to 4.2%, and in the high hospital tertile, 4.2% to 12.4%. Approximately, three‐quarters of hospitals were non‐academic centers, with the majority located in the South or Midwest (Table 1). When compared to high tertile hospitals, low tertile hospitals were significantly less likely to be academic and had fewer hospitals beds (Table 1).

**Table 1:** Hospital characteristics stratified by hospital tertiles of cardiac arrest patients

		All hospitals (n = 224) 0.9%‐12.4%	Low tertile hospitals (n = 74) 0.9%‐3.2%	Middle tertile hospitals (n = 75) 3.2%‐4.2%	High tertile hospitals (n = 75) 4.2%‐12.4%	*P*‐value
Region	West	32 (14.3)	8 (10.8)	11 (14.7)	13 (17.3)	0.58
	Northeast	12 (5.4)	3 (4.1)	3 (4.0)	6 (8.0)	—
	Midwest	74 (33.0)	24 (32.4)	29 (38.7)	21 (28.0)	—
	South	106 (47.3)	39 (52.7)	32 (42.7)	35 (46.7)	—
Hospital status	Academic^a^	51 (22.8)	13 (17.6)	10 (13.3)	28 (37.3)	<0.001
Total no. of hospital beds	—	338 (240, 522)	335 (226, 513)	306 (227, 430)	398 (294, 618)	0.028

Data presented as N (%) or median (25th, 75th percentiles).

a Council of Teaching Hospitals Membership.

Patient characteristics for the overall cohort are shown in Table 2. Median patient age was approximately 64 years (25th, 75th percentiles: 55,75), of whom approximately two‐third were male, almost three‐quarters had hypertension, diabetes was present in a third, with prior PCI and prior MI in about one quarter. When compared across hospital tertiles, risk factors were fairly evenly distributed, with no clinically significant differences among tertiles. However, there was an increase in the proportion of patients who had STEMI by hospital volume tertile, from 33.9% in the low to 43.0% in the high tertile hospitals. Concomitant with the increase in STEMI patients was an increase in the proportion who had cardiogenic shock, from 3.0% to 5.1% from the low to the high tertile. Among patients with cardiac arrest, most characteristics across hospital tertiles were similar, other than cardiogenic shock, which was higher in the high tertile group (37.9% in the low volume tertile compared to 45.3% in the high‐volume tertile) (Table S1).

**Table 2:** Baseline characteristics of all patients, stratified by hospital tertiles based on the proportion of patients who had cardiac arrest

	All patients (n = 252 882)	Low tertile hospitals (n = 85 147)	Middle tertile hospitals (n = 83 815)	High tertile hospitals (n = 83 290)	*P*‐value
Baseline characteristics					
Age (years)	64 (55, 75)	65 (55, 75)	65 (55, 75)	63 (54, 73)	<0.001
Male	65.4	64.5	65.3	66.4	<0.001
HTN	74.7	76.1	74.4	73.6	<0.001
DM	33.6	34.9	33.5	32.4	<0.001
Current/recent smoker (<1 year)	34.7	33.9	34.3	35.9	<0.001
Dyslipidemia	61.9	63.6	61.7	60.4	<0.001
PAD	10.0	10.5	10.1	9.4	<0.001
Cardiac history					
Prior Revasc	32.5	33.8	33.0	30.8	<0.001
Prior PCI	25.7	26.5	25.9	24.5	<0.001
Prior CABG	14.1	14.9	14.7	12.6	<0.001
Prior MI	25.8	27.3	25.7	24.6	<0.001
Prior HF	13.0	13.5	13.2	12.2	<0.001
Prior stroke	8.2	8.4	8.1	8.0	0.024
Presentation labs and characteristics					
Peak troponin ratio, xULN	58.3 (11.8, 277)	63.2 (12.7, 307.1)	57.0 (11.5, 270.0)	55.6 (11.3, 254.8)	<0.001
Initial CrCl, mL/min	82 (56, 112)	82 (55, 112)	81 (55, 111)	84 (58, 113)	<0.001
Initial Hb (g/dL)	14.0 (12.5,15.3)	14.0 (12.5,15.3)	14.0 (12.5, 15.3)	14.1 (12.5, 15.3)	<0.001
Cardiogenic shock	3.9	3.0	3.7	5.1	<0.001
Cardiac arrest	3.8	2.6	3.7	5.2	<0.001
Transferred in	32.9	31.3	32.5	35.0	<0.001
STEMI	37.6	33.9	35.9	43.0	<0.001

Abbreviations: CABG, coronary artery bypass graft; CrCl, creatinine clearance (calculated via Cockcroft‐Gault formula among non‐dialysis patients); DM, diabetes mellitus; Hb, hemoglobin; HF, heart failure; HTN, hypertension; MI, myocardial infarction; PAD, peripheral artery disease; PCI, percutaneous coronary intervention; revasc, revascularization; STEM, ST elevation myocardial infarction; ULN, upper limit of normal.

Data is reported either median (25th, 75th percentiles) or %.

The proportion of patients with STEMI who did not have contraindications who underwent primary PCI was similar in all patients and those with cardiac arrest (95.5%) (Tables 2 and 3). There was no clinical difference in the proportion of cardiac arrest patients who had primary PCI from hospitals with low‐ to high‐volume tertiles, which ranged from 96.2% to 95.3% (Table 3). The median door to balloon time in the cardiac arrest patients across hospital tertiles was similar. Of the NSTEMI patients, 51.5% underwent revascularization, where 40.9% had PCI and 11.9% had CABG. The rate of revascularization among NSTEMI patients was higher in the low volume tertile (54.2%) compared to with middle (50.5%) and high‐volume tertiles (50.9%) (Table S3).

**Table 3:** In‐hospital outcome events in cardiac arrest and non‐cardiac arrest patients stratified by hospital tertiles of cardiac arrest patients

	Low tertile hospitals		Middle tertile hospitals		High tertile hospitals	
	Cardiac arrest (n = 2211)	No cardiac arrest (n = 82 936)	Cardiac arrest (n = 3095)	No cardiac arrest (n = 80 720)	Cardiac arrest (n = 4376)	No cardiac arrest (n = 79 All other abbreviations can be found in Table 2544)
Death	29.8	3.2	31.2	3.6	31.4	3.3
Re‐MI	0.8	0.8	1.4	0.7	1.1	0.8
Major bleeding	27.2	6.4	25.8	6.9	27.8	6.8
Non‐CABG transfusion	12.6	5.0	10.3	5.1	11.6	4.7
Stroke	1.4	0.7	1.6	0.7	1.8	0.7

Abbreviation: CABG, coronary artery bypass graft; MI, myocardial infarction.

Pharmacologic treatment within 24 hours of hospital arrival was similar among cardiac arrest patients across volume tertiles, except for higher rates of ticagrelor and P2Y12 and lower rates of beta‐blocker use in the high‐volume tertile (Table S3).

### 3.1 In‐hospital outcomes and mortality

Patients with cardiac arrest were more likely to experience adverse in‐hospital events compared to those without cardiac arrest (Table 3). In‐hospital mortality in cardiac arrest patients was substantially higher than non‐cardiac arrest patients: 31% vs 3.3%. Unadjusted in‐hospital mortality for all MI patients (including those with and without cardiac arrest) was significantly lower in the lowest tertile (3.8%) when compared to the middle (4.6%) and high hospital tertile (4.7%)(*P* < 0.001); however, after adjustment for patient case mix the difference was no longer significantly different (OR = 0.93; 95% CI = 0.83, 1.05 for low compared with high tertile and OR = 1.02; 95% CI = 0.90, 1.16 for middle compared with high tertile).

## 4 DISCUSSION

We found that the overall proportion of contemporary MI patients surviving to hospital admission who had cardiac arrest was low, accounting for <4% of all MI patients. After adjustment using a model that included patient‐level variables and hospital characteristics, overall MI in‐hospital mortality did not differ significantly between hospitals that had low, intermediate, and high proportions of cardiac arrest MI patients.

We found that increasing hospital proportions of MI patients who had cardiac arrest did not affect overall in‐hospital risk‐adjusted mortality. There are number of potential reasons for our findings. First, the hospital proportion of patients with MI who had cardiac arrest was relatively small, accounting for a median of only 3.7% of all MI patients. Even in the high tertile, cardiac arrest patients accounted for 4.2% to 12.4% of all MI patients. The small proportion of patients appear to have been insufficient to affect overall reported mortality. Second, care processes and treatments were very similar among the different tertiles of cardiac arrest patients, and were similar to that of patients without cardiac arrest. For example, among STEMI patients with cardiac arrest who were considered appropriate candidates, more than 94% underwent primary PCI, and door to balloon times were similar in the low medium and high tertile (Tables S2 and S3). The NSTEMI patients also had high rates of coronary angiography, with many undergoing revascularization. Third, guideline recommended medical treatment within the first 24 hours of hospitalization was generally similar across tertiles.

### 4.1 Implications

The ACC^15^ and American Heart Association^16^, ^17^ and currently recommend regionalized patient care for patients resuscitated from out‐of‐hospital cardiac arrest at hospitals that are designated as cardiac resuscitation centers. Prior studies have demonstrated that regionalization of care for cardiac arrest patients has improved outcomes, whether done at the city^18^, ^19^, ^20^, ^21^ or state^22^, ^23^ level.

One concern is that care for cardiac arrest patients at specialized centers will adversely affect their reported mortality.^6^, ^24^, ^25^ For example, Peberdy et al^6^ attempted to estimate how increasing numbers of cardiac arrest patients with STEMI would affect hospital mortality. Using estimates of 100 STEMI patients a year, a 5% in‐hospital mortality for non‐cardiac arrest and a 50% for cardiac arrest patients, they calculated that an additional 10 STEMI cardiac arrest patients would almost double mortality from 5% to 9.1%. However, there was no risk adjustment. In contrast, our data indicate that risk standardized mortality for hospitals was similar regardless of the proportion of cardiac arrest patients admitted, suggesting that hospitals participating in regional systems of STEMI care are unlikely to be adversely penalized. This is important concept given that early reperfusion therapy is considered a Class I indication,^15^ and is now considered an MI performance measure.^26^ Our data are consistent with other studies indicating that risk adjustment appears adequate in most cases, as exclusion of patients with cardiac arrest^27^, ^28^ or cardiogenic shock, another high‐risk group^29^ did not significantly change reported mortality.

## 5 LIMITATIONS

Because we only included patients diagnosed with MI, we likely underestimated the total number of patients with cardiac arrest seen at each institution and therefore may not accurately assess the hospital's capability of treating cardiac arrest patients. Another potential limitation is the relatively low rate of cardiac arrest patients who were diagnosed with NSTEMI, which can result from difficulties in diagnosis. Troponin elevations are seen in almost all patients who have cardiac arrest^30^; whether this represents global myocardial damage or that related to MI can be difficult to determine in the absence of other confirmatory findings, such as angiography, or the ability to assess historical factors in patients who die before awakening. Finally, varying definitions of cardiac arrest can make it different to compare outcomes across studies. The definition used in the present study required either CPR or cardioversion, but did not require coma, which likely resulted in a lower risk patient population.

Because data used for our study are self‐reported by hospitals, there is a potential for data error. However, registry data collection utilizes consistent and frequent data quality algorithms that require predetermined levels of completeness and consistency before submission. Sites are provided reports to spur iterative data quality improvement, and annual audits are conducted in randomly selected hospitals, with a high degree of agreement.^11^ Although the mortality model that we used did not include cardiac arrest, the risk is partially accounted for by the high proportion of cardiogenic shock in cardiac arrest patients.^1^ We did not have information on the frequency of therapeutic hypothermia use, the initial rhythm, total arrest time or pre‐hospital treatments in cardiac arrest patients, or the volume of non‐MI cardiac arrest patients. We had limited hospital characteristics other than the ones included in our report; thus, were unable to further characterize hospital infrastructure, resources, and staffing. The ACTION Registry does not capture post‐discharge outcomes; therefore, we could not study long‐term mortality rates. Additional studies comparing mortality models that use clinical and administrative data in high risk patient populations may be useful.

## 6 CONCLUSIONS

After adjustment, in‐hospital mortality among all MI patients did not differ significantly between hospitals that had increaed proportions of cardiac arrest MI patients. Our data indicate that for most hospitals, overall adjusted MI mortality is unlikely to be adversely affected by treating cardiac arrest patients with MI. This analysis supports efforts to develop regional, high‐volume cardiac arrest centers.

## CONFLICTS OF INTEREST

The authors declare no potential conflict of interests.

## Supporting information


**Table S1** Patient baseline characteristics stratified by hospital tertiles of cardiac arrest patients among MI cardiac arrest patients
**Table S2** Initial treatments for all patients stratified by hospital tertiles of cardiac arrest patients
**Table S3** Initial treatments of cardiac arrest patients only, stratified by hospital tertiles
