# Supplementary material for: Association of acute myocardial infarction cardiac arrest patient volume and in‐hospital mortality in the United States: Insights from the National Cardiovascular Data Registry Acute Coronary Treatment And Intervention Outcomes Network Registry
Source: Clin Cardiol. 2019 Feb 7;42(3):352–7. doi: 10.1002/clc.23146 (PMC6712341; doi:10.1002/clc.23146)
Supplement: Supplementary file 1 — Table S1 Patient baseline characteristics stratified by hospital tertiles of cardiac arrest patients among MI cardiac arrest patients Table S2 Initial treatments for all patients stratified by hospital tertiles of cardiac arrest patients Table S3 Initial treatments of cardiac arrest patients only, stratified by hospital tertiles [file CLC-42-352-s001.docx]

Supplemental Table 1 Patient baseline characteristics stratified by hospital tertiles of cardiac arrest patients among MI cardiac arrest patients

|  | **Cardiac arrest patients**  **(n=9,682)** | **Low Tertile Hospitals**  **(n=2,211)** | **Middle Tertile Hospitals**  **(n=3,095)** | **High Tertile Hospitals**  **(n=4,376)** |
| --- | --- | --- | --- | --- |
| **Baseline characteristics** | | | | |
| **Age, years** | 62 (53, 71) | 62 (54, 72) | 62 (54, 71) | 61 (53, 70) |
| **Male** | 72.5 | 72.0 | 72.4 | 72.9 |
| **HTN** | 64.9 | 66.8 | 64.6 | 64.2 |
| **DM** | 25.7 | 26.1 | 26.3 | 25.1 |
| **Current/recent smoker (<1 year)** | 40.8 | 41.3 | 41.7 | 39.8 |
| **Dyslipidemia** | 49.1 | 52.2 | 49.7 | 47.1 |
| **PAD** | 8.1 | 8.6 | 8.2 | 7.6 |
| **Prior Revascularization** | 23.3 | 25.4 | 24.1 | 21.7 |
| **Prior PCI** | 17.8 | 19.1 | 18.6 | 16.5 |
| **Prior CABG** | 9.5 | 10.3 | 9.7 | 8.8 |
| **Prior MI** | 19.6 | 21.6 | 20.7 | 17.7 |
| **Prior HF** | 10.9 | 10.7 | 10.8 | 11.2 |
| **Prior Stroke** | 6.8 | 7.3 | 6.3 | 6.9 |
| **Presentation labs and characteristics** | | | | |
| **Peak Troponin ratio, xULN** | 167 (36.5, 700) | 245.0 (52.5, 1048.3) | 185.8 (38.6, 638.7) | 130.2 (29.0, 590.2) |
| **Initial CrCl, ml/min** | 76 (55, 100) | 75 (54, 98) | 75 (54, 99) | 77 (56, 101) |
| **Initial Hb, g/dL** | 14.0 (12.5,15.3) | 14.1 (12.5,15.3) | 14.0 (12.5, 15.4) | 14.0 (12.5, 15.3) |
| **Cardiogenic Shock** | 41.6 | 37.9 | 38.9 | 45.3 |
| **Transferred In** | 36.8 | 37.7 | 37.3 | 36.0 |
| **STEMI** | 73.2 | 72.3 | 71.7 | 74.8 |

Data is reported either median (25^th^, 75^th^ percentiles) or %. All other abbreviations can be found in Table 2.

Supplemental Table 2 Initial treatments for all patients stratified by hospital tertiles of cardiac arrest patients

|  | **All Patients**  **(n=252,882)** | **Low Tertile Hospitals**  **(n=85,147)** | **Middle Tertile Hospitals**  **(n=83,815)** | **High Tertile Hospitals**  **(n=83,290)** |
| --- | --- | --- | --- | --- |
| **Initial pharmacologic treatments within 24 hours of hospital arrival** | | | | |
| **Aspirin** | 98.4 | 98.6 | 98.4 | 98.3 |
| **P2Y_12_ receptor inhibitor** | 66.4 | 62.4 | 65.5 | 71.3 |
| **Clopidogrel** | 51.0 | 48.2 | 51.2 | 53.5 |
| **Prasugrel** | 14.8 | 14.3 | 13.8 | 16.2 |
| **Ticagrelor** | 14.1 | 10.6 | 15.0 | 16.7 |
| **Beta blocker** | 86.7 | 87.5 | 86.7 | 85.8 |
| **ACEI or ARB** | 47.6 | 47.8 | 46.1 | 48.8 |
| **Statin** | 69.7 | 69.7 | 68.0 | 71.3 |
| **Any heparin** | 85.7 | 86.7 | 85.3 | 84.9 |
| **Angiographic Information** | | | | |
| **Cath** | 88.9 | 88.3 | 87.9 | 90.4 |
| **Primary PCI* (STEMI)** | 95.5 | 96.4 | 94.8 | 95.3 |
| **D2B time, min (STEMI)** | 55 (41, 69) | 56 (41, 69) | 56 (42,69) | 55 (41,69) |
| **Time to Cath, hrs (NSTEMI)** | 5.3 (0.9, 24.0) | 8.7 (0.9, 26.9) | 6.2 (0.9, 23.9) | 2.8 (0.8, 21.5) |
| **Revascularization (NSTEMI)** | 61.2 | 61.3 | 60.4 | 62.0 |
| **PCI (NSTEMI)** | 49.9 | 49.5 | 49.1 | 51.5 |
| **Time to PCI, hrs (NSTEMI)** | 20.0 (8.5, 37.0) | 21.0 (9.4, 40.1) | 20.0 (8.7, 35.9) | 19.0 (7.5, 34.1) |
| **CABG (NSTEMI)** | 12.0 | 12.5 | 12.1 | 11.2 |

Data is reported either median (25^th^, 75^th^ percentiles) or %. *Primary PCI among reperfusion candidates not having facilitated or rescue PCI. Abbreviations: ACE = angiotensin converting enzyme; ARB = angiotensin II receptor blocker; Cath=cardiac catheterization; D2B=Door to balloon; NSTEMI=non-ST elevation myocardial infarction; All other abbreviations can be found in Table 2.

Supplemental Table 3 Initial treatments of cardiac arrest patients only, stratified by hospital tertiles

|  | **Cardiac arrest Patients**  **(n=9,682)** | **Low Tertile Hospitals**  **(n=2,211)** | **Middle Tertile Hospitals**  **(n=3,095)** | **High Tertile Hospitals**  **(n=4,376)** |
| --- | --- | --- | --- | --- |
| **Initial pharmacologic treatments within 24 hours of hospital arrival** | | | | |
| **Aspirin** | 92.6 | 93.2 | 92.2 | 92.5 |
| **P2Y_12_ receptor inhibitor** | 66.0 | 64.3 | 63.9 | 68.4 |
| **Clopidogrel** | 49.1 | 49.4 | 48.4 | 49.3 |
| **Prasugrel** | 16.0 | 15.6 | 16.0 | 16.3 |
| **Ticagrelor** | 18.5 | 14.1 | 18.8 | 20.5 |
| **Beta blocker** | 69.0 | 71.7 | 70.5 | 66.7 |
| **ACEI or ARB** | 28.7 | 27.5 | 27.4 | 30.1 |
| **Statin** | 52.2 | 51.7 | 49.0 | 54.8 |
| **Any heparin** | 80.9 | 81.1 | 81.0 | 80.8 |
| **Angiographic Information** | | | | |
| **Cath** | 88.1 | 87.5 | 87.0 | 89.2 |
| **Primary PCI* (STEMI)** | 95.5 | 96.2 | 95.2 | 95.3 |
| **D2B, min (STEMI)** | 54 (39, 67) | 52 (36, 65) | 55 (39, 67) | 54 (41, 68) |
| **Time to cath, hrs (NSTEMI)** | 1.0 (0.6, 1.6) | 1.0 (0.7, 1.6) | 0.9 (0.6, 1.6) | 1.0 (0.6, 1.5) |
| **Revascularization (NSTEMI)** | 51.5 | 54.2 | 50.5 | 50.9 |
| **PCI (NSTEMI)** | 40.9 | 42.4 | 39.5 | 41.1 |
| **Time to PCI, hrs (NSTEMI)** | 4.0 (1.7, 40.9) | 3.8 (1.7, 41.9) | 3.8 (1.7, 43.2) | 4.1 (1.8, 35.3) |
| **CABG (NSTEMI)** | 11.9 | 13.4 | 12.3 | 10.8 |

Data is reported either median (25^th^, 75^th^ percentiles) or %. *Primary PCI among reperfusion candidates not having facilitated or rescue PCI. All other abbreviations can be found in Table 2 and supplemental Table 2.
